# Fungal–Algal Association Drives Lichens’ Mutualistic Symbiosis: A Case Study with *Trebouxia*-Related Lichens

**DOI:** 10.3390/plants12173172

**Published:** 2023-09-04

**Authors:** Ya-Bo Zuo, Da-Yong Han, Yan-Yan Wang, Qiu-Xia Yang, Qiang Ren, Xin-Zhan Liu, Xin-Li Wei

**Affiliations:** State Key Laboratory of Mycology, Institute of Microbiology, Chinese Academy of Sciences, Beijing 100101, China

**Keywords:** *Trebouxia*, lichen, symbiosis, association, coevolution

## Abstract

Biotic and abiotic factors influence the formation of fungal–algal pairings in lichen symbiosis. However, the specific determinants of these associations, particularly when distantly related fungi are involved, remain poorly understood. In this study, we investigated the impact of different drivers on the association patterns between taxonomically diverse lichenized fungi and their trebouxioid symbiotic partners. We collected 200 samples from four biomes and identified 41 species of lichenized fungi, associating them with 16 species of trebouxioid green algae, of which 62% were previously unreported. The species identity of both the fungal and algal partners had the most significant effect on the outcome of the symbiosis, compared to abiotic factors like climatic variables and geographic distance. Some obviously specific associations were observed in the temperate zone; however, the nestedness value was lower in arid regions than in cold, polar, and temperate regions according to interaction network analysis. Cophylogenetic analyses revealed congruent phylogenies between trebouxioid algae and associated fungi, indicating a tendency to reject random associations. The main evolutionary mechanisms contributing to the observed phylogenetic patterns were “loss” and “failure to diverge” of the algal partners. This study broadens our knowledge of fungal–algal symbiotic patterns in view of *Trebouxia*-associated fungi.

## 1. Introduction

Lichens are vital constituents of fungi, encompassing approximately 20% of known fungal species [1]. Throughout the evolutionary history of Ascomycota and Basidiomycota, lichenization events have occurred independently on several occasions [2,3]. The unique mutualistic symbiosis of lichens involves fungi, green microalgae, or cyanobacteria [4,5], enabling their predominant presence in diverse and extreme environments, including polar, plateau, and desert regions [6]. Lichens also play a critical role in the early formation of terrestrial ecosystems [7].

As of now, there are approximately 20,000 accepted species of lichen-forming fungi (LFF) [1], whereas lichen-forming algae (LFA) are limited to around 200 species [8]. The composition of LFF and LFA in lichen symbiosis is not symmetrical [9]. However, the association between LFF and LFA is much more intricate than simply comparing the number of species. Various factors, both biotic and abiotic—such as the cophylogeny of LFF and LFA, geography, and the reproductive strategy of LFF—influence the formation of fungal–algal pairings in lichen symbiosis [10]. This indicates that both evolutionary and ecological processes contribute to the diversity of symbiotic associations [11].

The lichen associations are often described based on various degrees of availability, selectivity, and specifia between LFF and LFA. Specificity refers to a narrow taxonomic range of acceptable partners, while selectivity indicates the frequency of association with compatible partners [12,13]. In general, when compared to LFA–LFF associations, LFF–LFA associations exhibit higher specificity at any taxonomic level. For instance, at the order level, LFF like Arthoniales, Ostropales, Pyrenulales, and Trypetheliales, which are mainly found in tropical regions, preferentially associate with green algae from the Trentepohliales, while Lecanorales and Teloschistales are mainly associated with green algae from the Trebouxiales [14]. Generally, LFF genera and species tend to choose one specific genus of green algae or cyanobacteria and one specific species as their LFA [15,16,17]. However, LFF can range from being narrow specialists to generalists [18,19]. Most research on symbiont association patterns has been conducted using a specific lichen taxon as a model, with associations typically studied at the genus and family levels [5,8,15,20,21,22]. However, the association pattern between LFF and LFA is not well understood, particularly when distantly related fungi are considered.

Different lichen taxa often exhibit varied responses to diverse geographical factors concerning the LFF–LFA association [23,24]. Certain LFF are unaffected by geographical factors and maintain strong specificity to their associated LFA across significant global ranges. For instance, associations like the genus *Oropogon* with limited *Trebouxia* OTUs, *Omphalina* with *Coccomyxa*, and *Evernia mesomorpha* with *Trebouxia jamesii* s.l. demonstrate such patterns [15,25,26]. Alternatively, in some lichens, geographic distributions lead to distinct patterns of symbiosis, as observed in *Stereocaulon* and *Sticta* [21,22,27]. The ecological preference of LFA indirectly affects lichen distribution, where different LFF that associate with the same LFA generally exhibit similar ecological properties [28,29]. Asexual reproduction, through vegetative propagules such as isidia, soredia, and lobules (also called thallus fragmentation), allows lichens to colonize new habitats and co-disperse LFF and LFA (vertical transmission). Sexual reproduction, relying on the spores of LFF, leads to new symbiosis forming when suitable LFA are encountered (horizontal transmission) [30]. Asexual reproduction tends to structure stable mutualistic associations among lichens but may result in low LFA diversity. In contrast, sexual reproduction results in greater propagation distance and higher LFA diversity. However, existing studies either do not consider distantly related fungi or are limited to specific geographical zones. Consequently, the association pattern between LFF and LFA remains unclear when multiple types of geographical zones and different LFF orders are considered simultaneously.

Among the known lichen symbionts, approximately 50% to 70% form symbiotic relationships with green algae from the family Trebouxiaceae, with the genus *Trebouxia* being the most common LFA [8]. There is a growing interest in understanding the diversity and evolutionary relationships in *Trebouxia*-associated lichens [15]. Recent research has revealed a significant increase in candidate species lineages, ranging from 109 to 113, compared to the previously formally described 29 *Trebouxia* species, indicating an underestimation of *Trebouxia* diversity [5,31]. However, the samples in these studies from China were scarce, leaving room for the discovery of cryptic or unknown *Trebouxia* diversity, given its extensive ecogeographical range in the region. This creates an opportunity for an intensive study of *Trebouxia*-associated lichens’ diversity in China to comprehend their evolutionary patterns. Moreover, *Trebouxia*-associated lichens are found in various fungal orders and across different climatic zones.

In this study, we chose *Trebouxia*-associated lichen symbionts as a model and collected a total of 200 samples from different taxonomic levels (including orders) and distinct biomes, mainly from China. By delimiting the species of mycobionts and phycobionts, we aimed to explore the potential effects of various biotic and abiotic factors on the outcomes of the symbiosis, examine the interaction patterns between symbiotic associations across different ecoregions, and investigate their coevolutionary mechanisms.

## 2. Results

### 2.1. Molecular Sequence Data and Phylogenetic Analysis

In this study, a total of 1238 DNA sequences were generated, including 196 ITS, 188 nuLSU, 176 mtSSU, and 136 *RPB1* sequences from LFF and 186 ITS, 170 nuSSU, and 186 *rbc*L sequences from LFA (Table S1). The BLASTn analysis of the ITS sequences against the GenBank database revealed that the LFF sequences were classified into 41 species, 29 genera, 14 families, 8 orders, and 2 classes within Ascomycota (Table S1). Among these, 40 species belonged to Lecanoromycetes and 1 species belonged to Candelariomycetes, based on their high sequence identity with previously described species. The species delimitation was further confirmed using their phylogenetic topology based on the ITS dataset (Figure S1) and the four-locus concatenated phylogeny (Figure S2).

Based on the ITS sequence analysis using the BLASTn tool, all of the LFA were identified as belonging to the genus *Trebouxia*. The phylogenetic topology of *Trebouxia* was constructed using the combined ITS and *rbc*L sequences, incorporating sequences from the study by [5] (Figure S3). To confirm the species boundaries, three DNA species delimitation analyses (Generalized Mixed Yule Coalescent (GMYC), bPTP, and Automatic Barcode Gap Discovery (ABGD)) were employed (Figure S4). Sixteen species were recovered, of which ten were previously undescribed (Figures S3 and S5). Within the *Heterodermia japonica/H*. *speciosa* clade, different delimitation methods yielded varying results. Four or six *Trebouxia* species were recognized by GMYC (ITS), bPTP (ITS), ABGD (ITS + *rbc*L), and bPTP (ITS + *rbc*L), while only one species was recognized by GMYC (ITS + *rbc*L), ABGD (ITS), and the study by [5] (Figures S2, S4, and S5). Ultimately, the last delimitation was chosen, considering that some samples in this clade grow in the same locality, and even on the same bark, such as QJ2-1, QJ2-2, and QJ2-8. In another clade composed of 18 samples representing eight lichen species (*Bryoplaca tetraspora*, *Ochrolechia tartarea*, *Gondwania regalis*, *Megaspora verrucosa*, *Lecanora fuscobrunnea*, *Physcia caesia*, *Physcia dubia*, and *Rhizoplaca chrysoleuca*), all of the delimitation methods recognized only one *Trebouxia* species, except for GMYC (ITS), which recognized two (Figures S4 and S5). Consequently, the rule of majority was applied, and only one *Trebouxia* species was considered valid.

### 2.2. Mutualistic Association Patterns between LFF and LFA

**Figure 1:**
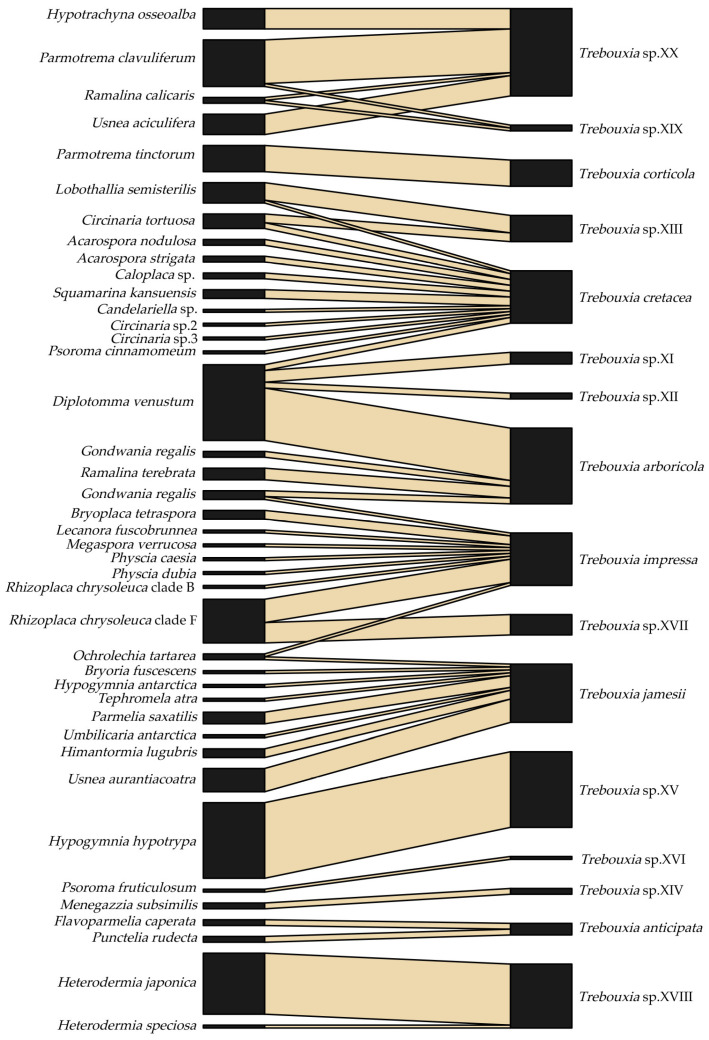
Mutualistic association patterns between lichen-forming fungi (LFF) and lichen-forming algae (LFA): The left and right columns represent the names of LFF and LFA, respectively. The lines connecting the LFF and LFA indicate the mutualistic associations. The width of the lines indicates the lichen sample numbers composed of LFF and LFA.

Fifty-one associations were observed between 41 LFF and 16 LFA (Figure 1; Table S1). At the class level, LFF species in Lecanoromycetes were associated with all 16 LFA *Trebouxia* species. At the order level, LFF orders with samples of more than two species were associated with multiple LFA species. For example, the species of Lecanorales, Caliciales, Teloschistales, and Pertusariales were associated with 10, 6, 3, and 2 LFA species, respectively. Similarly, at the family level, LFF species from Parmeliaceae, Megasporaceae, Teloschistaceae, and Physciaceae, with samples of more than two species, were associated with six, four, three, and two LFA species, respectively. At the genus level, *Circinaria* was the only genus with samples of more than two species, and it was associated with two LFA species. At the species level, each LFF species was associated with 1–4 LFA species. Among them, *Diplotomma venustum* showed the most potent selectivity but the poorest specificity to LFA, with an association ratio of 1 vs. 4.

Each *Trebouxia* species showed compatibility with 1–11 LFF species, and out of the 16 *Trebouxia* species, 11 were associated with more than one LFF species (Figure 1). Five LFA species—namely, *Trebouxia arboricola*, *T. cretacea*, *T. impressa*, *T. jamesii*, and *Trebouxia* sp. 10—were associated with 4–10 LFF species across various families, genera, and species. Among these, the first four LFA species were even associated with multiple LFF species distributed in different orders. *Trebouxia cretacea* exhibited the most robust selectivity but the poorest specificity to LFF, accepting 11 LFF species across two classes, seven orders, seven families, and eight genera.

Four pairs of associations between LFF and LFA displayed unique specificity, regardless of the sample source. These associations were *Hypogymnia hypotrypa* vs. *Trebouxia* sp. 5, *Menegazzia subsimilis* vs. *Trebouxia* sp. 4, *Parmotrema tinctorum* vs. *T. corticola*, and *Psoroma fruticulosum* vs. *Trebouxia* sp. 6. *Trebouxia* sp. 8 was found to form symbiotic relationships with two LFF species, namely, *Heterodermia japonica* and *H*. *speciosa*, but the majority of samples (95%; 21 out of 22) were associated with *H*. *japonica*. Most of these specific associations were observed in the temperate zone, indicating that these LFF–LFA associations tend to be more specific in warmer climates. The mutualistic associations corresponding to the four climate zones are summarized in Table 1. Interaction network analyses further revealed that the temperate network had relatively lower web connectance (0.17) and linkage density (1.83) but the highest nestedness (38.88), and it was segregated into more compartments (six) compared to the arid, polar, and cold networks.

### 2.3. Correlation among LFF, LFA, and Ecological Factors

**Figure 2:**
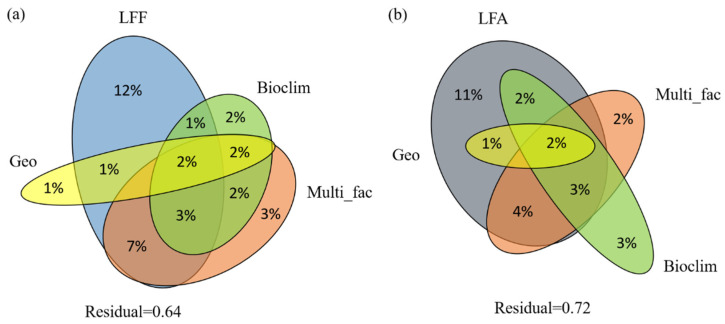
Venn diagram showing the results from variation partitioning analysis (VPA): (**a**) Partitioning variance of lichen-forming fungi (LFF), geographical distance (Geo), 19 bioclimatic variables (Bioclim), and other factors including lichen distribution types, reproduction modes, and host specificity (Multi_fac) onto photobiont diversity and distribution. (**b**) Partitioning variance of lichen-forming algae (LFA), geographical distance (Geo), 19 bioclimatic variables (Bioclim), and other factors including lichen distribution types, reproduction modes, and host specificity (Multi_fac) onto mycobiont diversity and distribution.

We performed variation partitioning analysis to assess the relative contributions of climatic variables, geographical distance, LFF or LFA, distribution types, reproductive modes, and host specificity to shaping the mutualistic association of lichens (Figure 2). For the LFA, climatic variables, geographical distance, LFF, and the other factors explained 36% of the variation (Figure 2a). The LFF explained the most significant proportion of the variation, with a 12% independent effect and 14% in combination with other variables. Distribution types, reproduction modes, and host specificity independently explained 3% of the variation, whereas 16% was shared with other variables. Geographical distance and climatic variables independently explained 1% and 2% of the variation, respectively, and 5% and 10% were shared with other variables, respectively. Alternatively, climatic variables, geographical distance, LFA, and other factors explained 28% of the variation of the LFF (Figure 2b). The LFA explained the most significant proportion of variation, with 11% independent effects and 12% in combination with other variables. The climatic variables independently explained 3% of the total variation and shared 7% with other variables, followed by 2% and 9% of variation explained by distribution types, reproduction modes, and host specificity independently and in combination with other variables, respectively. The geographical distance explained 3% when combined with other variables, while it explained 0% independently.

### 2.4. Cophylogenetic Analyses

The cophylogenetic analyses were conducted using the well-supported LFF and LFA phylogenies. The global-fit tests (ParaFit and PACo) provided evidence supporting overall congruence between the tree topologies of fungal hosts and *Trebouxia* species (ParaFitGlobal = 0.08, *p* = 0.001; PACo m^2^ global value = 2.73, *p* = 0; Figure S6). These results reject the null hypothesis of random association. Out of the 51 mutualistic associations studied, 16 (31.4%) showed significant contributions to ParaFitGlobal based on ParaFit1 values, and 18 associations (35.3%) were based on ParaFit2 values (*p* ≤ 0.05).

In Jane 4.0, most cost scenarios tested supported significant congruence between LFF and LFA phylogenies. The program’s reconstructed solution outperformed all random solutions except for costs H and Q (Table 2). Notably, among the significant reconstructions, cost regime B yielded the lowest overall cost. This regime suggested one cospeciation event, eight duplications, six duplications with host switches, ninety losses, and thirty-five failure-to-diverge events, all contributing to the congruent phylogenies between LFF and LFA (Figure S7). The roles of losses and failure-to-diverge events were found to be crucial in shaping the observed congruence, and interestingly, all of the cost regimes yielded the same solution regardless of the penalized values.

## 3. Discussion

Accurate species delimitation of symbiotic partners is crucial to avoid biased identity, which would mask interactions [20]. The ultrastructure of LFA was analyzed using low-temperature scanning electron microscopy, offering a new species identification strategy [32], which merits incorporation into the LFA integrative classification. In this study, we tentatively conducted phylogenetic analysis using the dataset of [5] as a backbone to construct the phylogeny of *Trebouxia* species. We employed three species delimitation approaches to define the algal species boundary, and the results were consistent with those of [5] (Figure S3). From this analysis, we identified 16 *Trebouxia* species, of which 10 (62.5%) are potentially new species to science, indicating an underestimation of cryptic *Trebouxia* species diversity. In dealing with inconsistent delimitation results for two species (*Heterodermia japonica* and *H*. *speciosa*), we ultimately adopted the minimum number of species, considering the habitat and the previous delimitation results [32], to avoid overestimating *Trebouxia* species diversity. Nevertheless, our findings strongly suggest a higher potential for diversity than currently identified.

Our data revealed that 32 (78%) of the 41 LFF species were associated with only one LFA species, whereas 11 (69%) of the 16 LFA species accepted more than one LFF species (Figure 1). This mutualistic association exhibits asymmetry, suggesting higher selectivity and weaker specificity from the perspective of LFA choosing LFF over the reverse. However, this result might have been partially influenced by the limited samples available for analysis. Previous studies on interaction networks have proposed the abundance–asymmetry hypothesis [33,34], which hypothesizes that the uneven distribution of fungal hosts and algal species contributes to this asymmetry. According to this hypothesis, species abundance plays a crucial role in determining the frequency and strength of interspecific interactions, resulting in an asymmetric structure when individuals interact randomly within a community [35]. Given the substantial difference in known species abundance between LFF and LFA [1,8], it is inevitable that this abundance disparity impacts the mutualistic association patterns observed. Additionally, the reproduction modes of LFF also influence the asymmetric mutualistic association patterns, as they exhibit both sexual and asexual reproduction modes [36]. Macrolichens, including most species in our study, typically exhibit asexual structures such as isidia and soredia [36]. These structures facilitate the dispersal of both LFF and LFA together, resulting in relatively low genetic diversity in both partners and high specificity in their reciprocal association [10]. The association pattern between LFF and LFA in asexual lichens differs from that observed in sexual lichens within the lichen genus *Cladonia* [10] However, when we consider a broader taxonomic scale in our study, the role of reproductive modes of LFF in shaping the LFF–LFA association patterns explains less than 2% of the variation. In mutualistic association, LFF tend to act as specialists. Alternatively, LFA species are seldom found in a free-living state and cannot survive independently once the lichens die. As a result, they tend to act as generalists in the mutualistic association chosen by LFF, particularly in harsh environments [37,38]. Similar patterns are observed in trentepohlioid algae-associated lichens, where closely related algae associate with distantly related mycobionts or with mycobionts from one family [39]. Nevertheless, it is essential to note that not all LFF play the same role as strict specialists, sometimes acting as generalists instead [40].

In our study, different symbiotic partners exhibited diverse association patterns across various climatic zones and ecoregions. Interaction network analyses showed that the cold and polar zones had higher web connectance compared to the temperate zone, indicating increased reciprocal specificity between partners in warmer regions, consistent with the findings of Singh et al. [41]. However, this is not a fully confirmed conclusion, because although the polar zone still had higher linkage density the cold zone had lower linkage density than the temperate zone, indicating that temperature is not the absolute factor shaping the LFF–LFA association. Moreover, the nestedness was the highest in temperate zone, which was an almost contrary result compared to the findings of Singh et al. [41]. It is interesting instead that there was the lowest nestedness in the arid zone, indicating drought might be an underestimated factor in structuring the specificity of LFF–LFA associations. The selection of LFA is also sometimes influenced by the LFF within a taxonomic range of acceptable partners, especially at the local scale [42]. Conversely, the patterns of LFF’s specificity toward LFA remain consistent across different ecoregions and climatic zones. In most cases, LFF maintain one-to-one relationships with LFA, except for a few instances, such as *Circinaria tortuosa*, *Diplotomma venustum*, *Gondwania regalis*, *Lobothallia semisterilis*, *Ochrolechia tartarea*, *Parmotrema clavuliferum*, and *Ramalina calicaris*, which associate with 2–4 LFA species. Among all of the mutualistic associations, the most distinct modularity is observed in *Hypogymnia hypotrypa*, where both LFF and LFA act as one-to-one specialists. This high reciprocal specificity is likely related to their vertical transmission through soredia and the limited niche of LFA. Similar patterns have also been found in *Evernia mesomorpha*, which belongs to the same family as *Hypogymnia hypotrypa* [16,26].

Our study revealed that symbiotic partners have the most significant impact on mutualism, with 11–12% independent effects and 12–14% in combination with other variables, which are lower than the corresponding values found in studies focusing on a single lichen genus, such as *Parmelia* [43]. The contribution of LFF and LFA to their respective partners is based on the genetic distance of their phylogenies. In the lichen genus *Cladonia*, the identity of LFF has a more pronounced impact on the genetic variation of LFA (*Asterochloris*) [44], leading to the occurrence of more new LFF–LFA associations. Hence, the effects of the symbiotic partners could be understood as their cophylogenetic trend, where the effect value is roughly equivalent to the degree of cophylogenetic evolution. Variation partitioning analyses demonstrated that geographical location (GPS) and 19 climate factors related to temperature and precipitation contributed no more than 3% to the variation. Similar findings have been observed in many other symbiosis analyses, where symbiotic partners primarily drive mutualism, while ecological mechanisms and geographical distributions such as extreme environments or cooler climate zones explain part of the variation [15,21,30]. However, within a specific taxonomic scale, ecological factors like climate exhibit a more significant impact on structuring LFF–LFA associations [43,45]. In the island radiation of LFF, macroclimate plays a more critical role in the LFF–LFA association than LFA [40].

There are still 64% and 72% unknown interpretations that need further exploration. One of these factors is historical climatological and geological processes, which have been shown to affect current species distribution patterns [46]. For example, the distribution of *Trebouxia* has been linked to climate change, which, in turn, might impact LFF and their nutritional dependence on LFA [24]. However, the extent of this factor’s effect was not known in this study. Moreover, the research model in this study involves *Trebouxia*-associated lichens from different genera, families, and orders, making the interactions more complex than those studied at the species or genus levels. Studies focusing on a single fungal genus could provide an explanation of the variation within one fungal genus that could be easily obtained [21,41].

Cophylogeny evaluates the dependency of two associated groups of organisms to investigate how ecological and evolutionary processes affect species diversification [47]. Our study rejected the null hypothesis of a random association between LFF and LFA, as previously reported by other studies [5,11,22,48], indicating a trend of congruence between *Trebouxia* algae and their associated LFF. Loss and failure of LFA to diverge played a much more critical role than cospeciation, duplication, and host switching in driving the coevolution in lichen mutualism (Figure S7), although some scientists did not consider coevolution [49]. It has been verified that cospeciation between symbionts seldom happens except for those with strictly vertical transmission, as opposed to reciprocal selection by mutualistic partners, such as lichens [50]. Host switches, failure of the LFA to diverge, and losses are commonly found to be the prevalent events shaping lichen symbionts, such as *Protoparmelia*–*Trebouxia*, *Sticta*–*Symbiochloris*, and *Cladonia*–*Asterochloris* [22,41,51]. The failure of the LFA to diverge with their fungal hosts is known to be responsible for the occurrence of generalist algal species [52]. This was demonstrated by one *Trebouxia* species being associated with multiple fungal species across different taxonomic levels in our study. Losses of LFA are a consequence of extinction or incomplete lineage sorting, which has been reported as another significant event during the coevolutionary process of lichens [41]. This is usually caused by the inability of the algae to parasitize the fungal hosts when the host speciates incipiently with a small population size [50,52]. Furthermore, this study’s evolutionary events leading to *Trebouxia*-associated symbionts did not correspond to the climate zones. In contrast, macroclimate may influence unique lichen association patterns, such as *Protoparmelia–Trebouxia* symbionts driven by different evolutionary mechanisms in different climatic regions [41].

Our study highlights the association between LFF and LFA in *Trebouxia*-related lichens. The sampling scope was not limited to species, genus, family, or order but covered a broader taxonomic scale, providing a comprehensive framework of lichen phylogeny. Additionally, our study included different climatic zones and ecological types, ranging from local to regional and global scales, rather than being restricted to a specific geographical area. Although the sample size of this study may not be very large and it may not present the complete picture of *Trebouxia*-related lichens, it still offers an overview of the LFF–LFA mutualistic associations on larger taxonomic and ecological scales. To gain a more comprehensive understanding of the coevolution of mutualistic associations, future explorations should consider incorporating more climate zones, ecological types, taxa, and larger sample sizes.

## 4. Materials and Methods

### 4.1. Taxon Sampling and Morphological Examination

**Figure 3:**
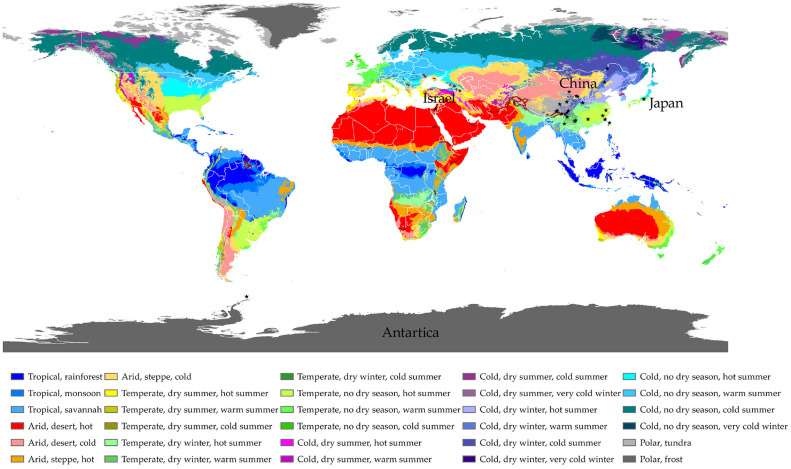
Collection sites of the lichen materials used in this study are marked by solid blue circles. The map was taken from [54]. The color scheme was adopted from [53].

Between 2014 and 2018, we collected 200 *Trebouxia* lichen samples from diverse geographical locations, including mainland (forest and sand desert) and Taiwan (forest) of China, Japan (forest), Israel (sand desert), and Antarctica (cold desert). These collections spanned four climate zones (B—Arid, C—temperate, D—cold, and E—polar) based on the Köppen–Geiger climate classification [53,54] (Figure 3, Table S2). The samples covered a wide range of taxonomical scales, representing different orders, families, genera, and species, mainly from the class Lecanoromycetes. Morphological and anatomical examinations were conducted using a MOTIC SMZ-168 stereomicroscope, a Leica M125 dissecting microscope equipped with a Leica DFC450 camera, and a Zeiss Axio Imager A2-M2 equipped with a Zeiss AxioCam MRC5 camera.

### 4.2. DNA Extraction, Amplification, and Sequencing

Samples’ DNA was extracted either fresh or frozen at −80 °C. Healthy fragments without any diseased spots were selected and surface-sterilized in 75% ethanol, 1% sodium hypochlorite, and 75% ethanol for 1 min, 2 min, and 0.5 min, respectively, followed by a rinse with sterile water for 5 min. Total genomic DNA was extracted from lichen thalli using a modified CTAB method [55]. For LFA, we amplified the nuclear internal transcribed spacer region (ITS), the nuclear small subunit ribosomal RNA gene (SSU), and the chloroplast ribulose-bisphosphate carboxylase-RuBisCO gene (*rbc*L). For LFF, four nuclear markers were amplified: ITS, the large nuclear subunit ribosomal RNA gene (nuLSU), the largest subunit of RNA polymerase II (RPB1), and the small mitochondrial subunit ribosomal RNA gene (mtSSU). PCR amplification was conducted in a 25 μL reaction volume, comprising 12.5 μL of 2 × Taq Mix DNA polymerase (CWBIO, China), 9.5 μL of ddH_2_O, 1 μL each (10 μM) of the primers (forward and reverse), and 1 μL of the DNA template. The primers used were obtained from various sources [56,57,58,59,60,61,62,63,64,65,66,67,68,69]. Detailed PCR conditions can be found in Table S3. The PCR products were visualized on 0.8% agarose gels, purified using a gel purification kit (Shanghai Huashun Bioengineering Corporation, China) according to the manufacturer’s instructions, and subsequently sequenced using an ABI 3730 XL Sequencer (Shanghai BioSune Corporation, China).

### 4.3. DNA Alignment and Phylogenetic Analysis

The DNA sequences were assembled and manually edited using the SeqMan module of the DNASTAR Lasergene Packages 7 (Madison, WI, USA). Each locus’s sequences were aligned separately using MAFFT v. 7.471 [70] with the G-INS-i algorithm. Gaps were treated as missing data, and ambiguously aligned regions were excluded using Gblocks v0.91b [71]. The sequence data were submitted to the GenBank database, and the accession numbers are provided in Table S2. Additionally, the sequence alignments were deposited at TreeBase (http://purl.org/phylo/treebase/phylows/study/TB2:S30069 (accessed on 16 January 2023)).

Individual gene topologies of LFA and LFF were reconstructed using the RAxML v.8 program [72], which was implemented on the CIPRES Science Gateway v.3.3 [73]. The reconstruction was performed with a GTRGAMMA model and 1000 bootstrap (BS) replicates to assess the branch support. Before concatenating the datasets, the individual gene topologies were carefully examined for congruence. A conflict was considered significant when two data partitions supported conflicting monophyletic groups with maximum-likelihood (ML) bootstrap values of ≥70% in both trees [74]. To achieve maximum phylogenetic resolution without evidence of conflicting nodes, independent gene datasets were combined.

Bayesian inference was carried out on the concatenated datasets of LFA and LFF using MrBayes v.3.2.7. [75] on the CIPRES Science Gateway v.3.3. The best-fitting models of nucleotide substitutions were selected based on the corrected Akaike information criterion (AICc), as suggested by jModelTest [76]. Two parallel Markov chain Monte Carlo (MCMC) runs were conducted, each utilizing four chains and running for 10,000,000 generations. Trees were sampled every 10,000th generation. After discarding the initial 25% as burn-in, a 50% majority-rule consensus tree was generated from the combined sample of both runs.

The phylogenetic trees resulting from the analysis were visualized using FigTree v.1.4.4 [77]. Clades with bootstrap support (BS) values of ≥70% and posterior probability values of ≥0.95 were considered to be significantly supported.

### 4.4. Species Delimitation

The identification of LFF was primarily based on DNA sequences, in conjunction with phenotypic examination. To identify the closest relatives of each strain, a BLAST search against the GenBank database was conducted using the BLASTn tool. The LFF strains were initially classified at the species level, mainly based on the threshold >98% sequence identity with the described species in the ITS region. A total of 1371 sequences of their closest relatives were downloaded from GenBank and used for assessing the phylogenetic positions of 1238 newly generated LFF sequences from this study. Both ML and Bayesian methods were employed to construct a four-locus phylogenetic tree in order to re-evaluate the phylogenetic relationships of LFF strains. For the identification of LFA and their phylogenetic positions, we integrated ITS and *rbc*L sequences from this study into the *Trebouxia* dataset from [5] for further phylogenetic analysis. The identity of the *Trebouxia* species was then evaluated using three species delimitation approaches: ABGD [78], a Bayesian implementation of the PTP approach (bPTP) [79], and the coalescent-based GMYC approach [80].

ABGD infers a model-based confidence limit for intraspecific divergence and identifies the barcode gap as the first significant gap beyond this limit to partition the data. The primary data partitions are then recursively split to obtain finer partitions until no further partitions can be detected [78]. For the ABGD analyses, separate analyses were performed for each dataset of ITS, SSU, and *rbc*L using the web interface (https://bioinfo.mnhn.fr/abi/public/abgd/abgdweb.html (accessed on 22 May 2023)). Genetic distances were calculated using the JC69 model, with a prior P ranging from 0.001 to 0.1 and a relative gap width ranging from 1.0 to 1.5. Regarding bPTP (Bayesian implementation of the PTP approach) analyses, unrooted phylogenetic trees inferred by ML were used. The analyses were run on the bPTP web server (http://species.h-its.org/ptp/ (accessed on 31 December 2022)) with 200,000 generations, a burn-in of 0.1, and a thinning of 100. Both ML and Bayesian solutions were examined. For the GMYC analyses, ultrametric trees were estimated from the combined sequence dataset using the BEAST v2.6.3 program [81]. MCMC chains were run for 15 million generations under the coalescent model with a constant population size and a constant clock as the tree prior. Chain mixture and convergence were evaluated in TRACER v.1.7.2 [82] after discarding 10% of the samples as burn-in. An effective sample size value greater than 200 was considered to be a good indicator. The consensus tree was generated using TreeAnnotator 1.8.2 [81] after discarding the first 3000 trees. The GMYC analysis was executed under the single-threshold model using the SPLITS package [80], available for R 4.0.5 [83].

### 4.5. Interaction Network Analyses

The mutualistic association network of *Trebouxia* species and their associated fungal symbionts (Table S4) was visualized in R using the function *plotweb* from the bipartite package, based on the species trees [84]. Bipartite network analyses were conducted to assess the complexity of the interactions using five commonly used qualitative indices: connectance, linkage density, links per species, number of compartments, and nestedness. These analyses were performed using the function *networklevel* from the R package bipartite [84,85].

### 4.6. Variation Partitioning Analysis

The variation in LFA and LFF diversity was analyzed using variation partitioning analysis based on redundancy analysis. The analysis aimed to examine the relative effects of several factors, including climatic variables obtained from the GBIF website and distribution literature related to the lichen species used in this study, geographical distance, the symbiotic partner, and other factors, such as distribution types, reproduction modes (sexual—fertile; asexual—soredia/isidia/lobules), and host specificity (i.e., the number of LFF species associating with the LFA, or vice versa; Table S5). The variation partitioning analysis was conducted using the *varpart* function in the vegan package [86]. The phylogenetic distances of LFA or LFF were calculated using the Patristic software, and the first 10 PCoA (principal coordinate analysis) axes were utilized as response variables [87]. Nineteen climatic variables for the 200 samples (Table S5) were obtained from the WorldClim database (www.worldclim.org (accessed on 20 June 2022)) with a grid cell resolution of 2.5 min using the software DIVA-GIS 7.5 [88]. These 19 environmental variables underwent forward selection using the function *RDA* in vegan with the distribution of LFA and LFF. Geographical distance values (i.e., latitude and longitude) were transformed into principal coordinates of neighbor matrix (PCNM) vectors representing geographical distances at various spatial scales. PCNM vectors were calculated based on the pairwise geographical distances using the function *pcnm* in the vegan package. All four PCNMs were used for the analysis. Additionally, a presence/absence matrix of 30 variables, including distribution types, reproduction modes, and host specificity, was combined as a mixed factor. Similar to the climatic variables, forward selection was applied to the distribution of LFA and LFF.

### 4.7. Cophylogenetic Analyses

To assess the cophylogenetic patterns between LFF and LFA, three methods were used: ParaFit [89], the Procrustean Approach to Cophylogeny (PACo) [90], and Jane 4.0 [91]. One sample corresponding to each LFF–LFA association was chosen for the cophylogenetic analysis to evaluate potential patterns of coevolution and congruence between the two associated groups of organisms.

Distance-based analysis was conducted to assess the overall congruence between host and parasite phylogenies, alongside quantification of the relative contribution of individual host–parasite associations to the overall congruence. The ParaFit method was implemented using the R package APE [92]. The ParaFitGlobal statistic, representing the fit between parasite and host phylogenies, was computed, and its statistical significance was tested through 999 permutations to determine whether the parasites were randomly associated with their hosts. If the observed ParaFitGlobal statistic exceeds the randomized statistic in more than 95% of cases, the null hypothesis of independent evolution can be rejected, indicating a significant congruence between host and parasite phylogenies. To identify the contribution of individual host–parasite associations, the ParaFitLink1 and ParaFitLink2 tests were employed. A significant link suggests that a particular host–parasite association contributes to the global congruence between the host and parasite trees. Furthermore, PACo analysis was performed using the R packages APE and VEGAN [86]. This analysis assessed the dependence of algal phylogeny on the fungal host phylogeny through 100,000 permutations. The contribution of each host–parasite association to the global fit was measured using jackknife estimation of their respective squared residuals and the confidence intervals associated with each host–parasite association.

The event-based analyses were conducted using the Jane 4.0 software (Claremont, CA, USA), which supports polytomies and accommodates parasites with multiple hosts. The topology-based program assigns costs to five coevolutionary events: cospeciation, duplication, host switching, loss/extinction, and failure to diverge [91]. Estimating the relative cost of events is challenging; hence, a default event cost scheme was employed (cospeciation = 0, duplication = 1, host switching = 2, loss/extinction = 1, failure to diverge = 1). Additionally, 16 other cost regimes were selected, derived from the default regime, to test the overall costs of reconstructions in Jane 4.0. All regimes were analyzed with a population size of 23, following the recommendation of [91]. The number of generations was set to 45. To evaluate the statistical significance of the reconstructions, 100 random tip mapping permutations were performed.

## Figures and Tables

**Table 1:** The interaction-network-level parameters.

	Arid Zone	Cold Zone	Polar Zone	Temperate Zone
Connectance	0.30	0.44	0.22	0.17
Links per species	1.00	0.67	0.87	0.71
Linkage density	4.60	1.50	3.70	1.83
Nestedness	18.68	24.21	37.39	38.88
Number of compartments	1	2	3	6

**Table 2:** Cophylogenetic analysis results with an event-based approach using Jane 4.0 with different cost regimes.

Cost Regime	C-D-D&S-L-FD ^a^	C	D	D&S	L	FD	Total Cost
A	0,1,2,1,1	1	8	6	91	35	146
B	0,1,2,1,−1	1	8	6	90	35	75
C	0,2,2,1,1	1	8	6	89	35	152
D	0,0,2,1,1	1	9	5	91	35	136
E	0,1,3,1,1	1	9	5	91	35	150
F	0,1,1,1,1	0	8	7	88	35	138
G	0,1,2,2,1	0	8	7	90	35	237
H	0,1,2,0,1 *	2	13	0	113	35	48
I	0,1,2,1,2	1	8	6	89	35	179
J	0,1,2,1,0	1	8	6	90	35	110
K	1,1,1,1,1	0	8	7	88	35	138
L	1,1,2,1,1	0	8	7	88	35	145
M	1,0,0,1,1	0	7	8	90	35	125
N	−1,1,2,1,1	1	8	6	89	35	143
O	2,1,1,1,1	0	8	7	88	35	138
P	2,1,1,1,0	0	8	7	88	35	103
Q	2,1,1,0,0 *	0	8	7	108	35	15

Notes: ^a^ Order of cost regimes from left to right is C (cospeciation), D (duplication), D&S (duplication and host switch), L (loss/sorting), and FD (failure to diverge). * This cost regime is not significantly lower than random reconstruction.

## Data Availability

The DNA sequence alignments were deposited at TreeBase (http://purl.org/phylo/treebase/phylows/study/TB2:S30069 (accessed on 16 January 2023)).

## Supplementary Materials

The following supporting information can be downloaded at: https://www.mdpi.com/article/10.3390/plants12173172/s1, Figure S1: Phylogeny of 41 species of lichen-forming fungi based on Randomized Axelerated Maximum Likelihood (RAxML) analysis using the internal transcribed spacer region (ITS) sequences. The species names and accession numbers of type strains are marked in red. The number in each node represents bootstrap support (BS) and posterior probability (PP). BS values of ≥70 and PP values of ≥0.95 are plotted on the branches. Scale in 0.02 substitutions per site. Figure S2: Phylogeny of lichen-forming fungi based on the Randomized Axelerated Maximum Likelihood analysis of the concatenated four-locus dataset including the internal transcribed spacer region (ITS), the large nuclear subunit ribosomal RNA gene (nuLSU), the small mitochondrial subunit ribosomal RNA gene (mtSSU), and the largest subunit of RNA polymerase II (RPB1) sequences. The number in each node represents bootstrap support (BS) and posterior probability (PP). BS values of ≥70 and PP values of ≥0.95 are plotted on the branches. Scale in 0.02 substitutions per site. Figure S3: Phylogeny of lichen-forming algae *Trebouxia* species based on the Randomized Axelerated Maximum Likelihood (RAxML) analysis of a concatenated two-locus dataset including internal transcribed spacer region (ITS) and chloroplast ribulose-bisphosphate carboxylase-RuBisCO (*rbc*L) gene sequences, with reference sequences from the studies of [5,93,94,95,96] marked in black. The number in each node represents bootstrap support (BS). BS values of ≥70 are plotted on the branches. Scale in 0.05 substitutions per site. Figure S4: Comparison of different *Trebouxia* species delimitations resulting from three species delimitation approaches, i.e., Automatic Barcoding Gap Discovery (ABGD), a Bayesian implementation of the PTP (bPTP) [79], and the coalescent-based General Mixed Yule Coalescent (GMYC), along with the species scenarios proposed by [5]. The phylogeny was constructed by Randomized Axelerated Maximum Likelihood (RAxML) analysis based on the combined internal transcribed spacer region (ITS) and chloroplast ribulose-bisphosphate carboxylase-RuBisCO gene (*rbc*L) gene sequences. Scale in 0.02 substitutions per site. The numbers in the seven columns represent the species numbers defined by different methods; Figure S5: Phylogeny of lichen-forming algae *Trebouxia* species based on the Randomized Axelerated Maximum Likelihood (RAxML) analysis of a concatenated three-locus dataset including internal transcribed spacer region (ITS), large nuclear subunit ribosomal RNA gene (nuSSU), and chloroplast ribulose-bisphosphate carboxylase-RuBisCO (*rbc*L) sequences. The number in each node represents bootstrap support (BS) and posterior probability (PP). BS values of ≥70 and PP values of ≥0.95 are plotted on the branches. Scale in 0.01 substitutions per site; Figure S6: Boxplot of the jackknifed squared residuals, with upper 95% confidence intervals associated with each host–symbiont link from Procrustean Approach to Cophylogeny (PACo) analysis. Asterisks on the top of the bars indicate significant congruence, as supported by ParaFit. Figure S7: The least costly cophylogenetic scenario between lichen-forming algae (LFA) and their lichen-forming fungi (LFF) hosts, reconstructed using Jane 4.0. The cost regime settings were as follows: cospeciation = 0, duplication = 1, duplication with host switch = 2, losses = 1, and failures to diverge = −1, corresponding to cost regime E (Table 2). Black branches represent the LFF host phylogeny, and blue branches represent the LFA parasite phylogeny. The LFF names are in bold. Yellow and red solid circles represent duplications, dashed lines with purple circles represent losses, and dented lines with black asterisks represent failures to diverge. Habitat information is provided with the LFF species. AR: arctic/alpine or boreal zone, TM: temperate zone, STR: subtropical zone, TR: tropical zone. Table S1: The BLASTn results for LFF strains. The species names in red are included as reference species in the ITS phylogeny. Table S2: Lichen samples used in this study, including voucher information and GenBank accession numbers. Table S3: Molecular markers and primer sequences used in this study. Table S4: The climate type of lichen distributions. Table S5: The information on geographical distance, bioclimatic variables, and other factors of lichen-forming fungi and algae.
