# Supplementary material for: Fungal–Algal Association Drives Lichens’ Mutualistic Symbiosis: A Case Study with Trebouxia-Related Lichens
Source: Plants (Basel). 2023 Sep 4;12(17):3172. doi: 10.3390/plants12173172 (PMC10490544; doi:10.3390/plants12173172)
Supplement: Supplementary file 1 [file plants-12-03172-s001.zip › plants-2419170 Figures S1-S7.pdf]

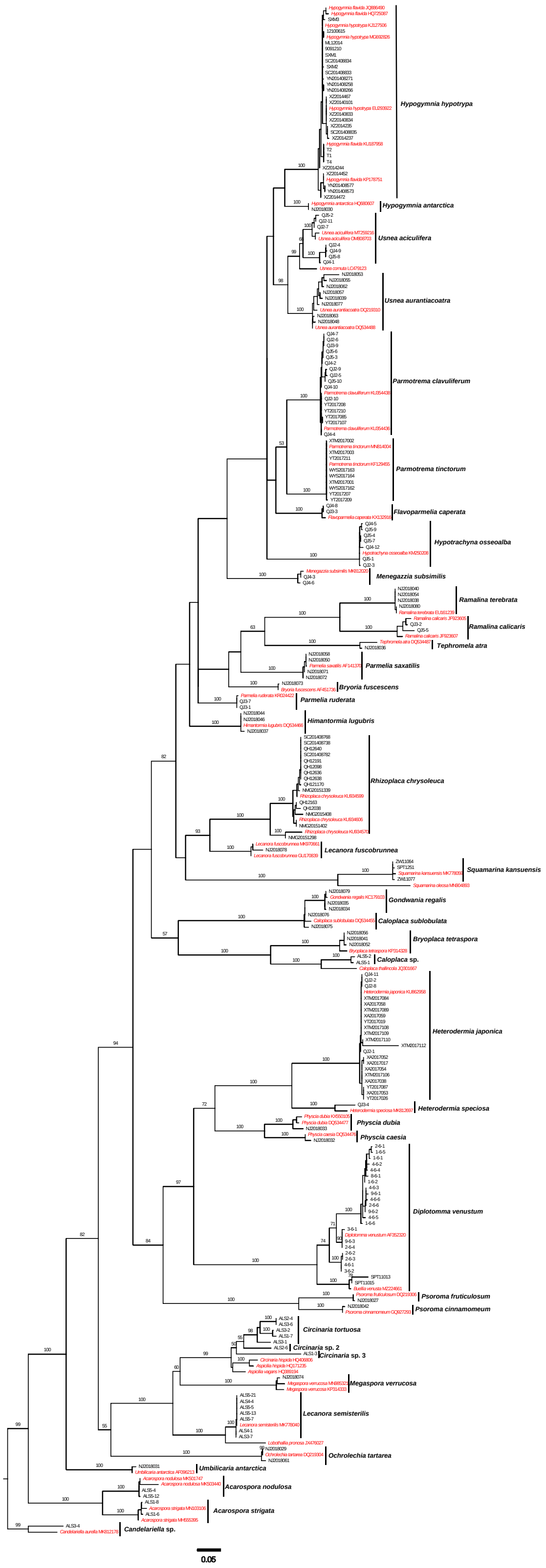

Figure S1. Phylogeny of 41 species of lichen-forming fungi based on Randomized Axelerated Maximum Likelihood (RAxML) analysis using the internal transcribed spacer region (ITS) sequences. The species names and accession numbers of type strains are marked in red. The number in each node represents bootstrap support (BS) and posterior probability (PP). BS values of ≥70 and PP values of ≥0.95 are plotted on the branches. Scale in 0.02 substitutions per site.

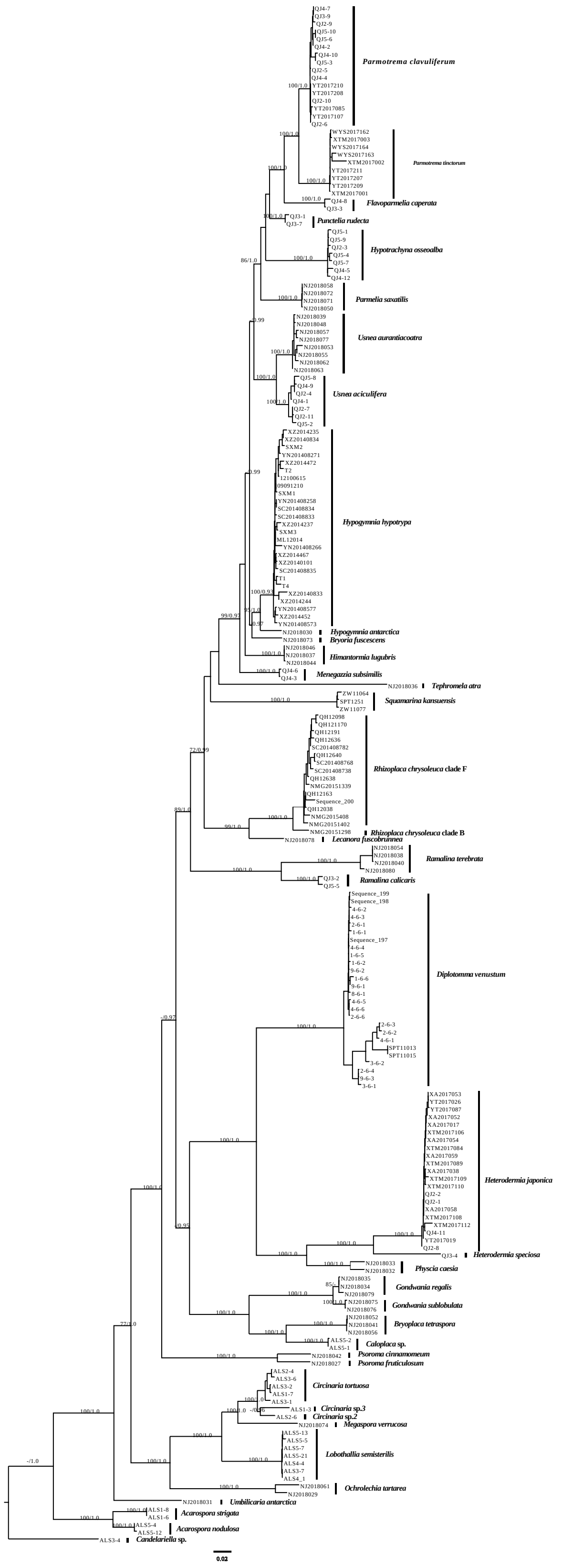

Figure S2. Phylogeny of lichen-forming fungi based on the Randomized Axelerated Maximum Likelihood analysis of the concatenated four-locus dataset including the internal transcribed spacer region (ITS), the large nuclear subunit ribosomal RNA gene (nuLSU), the small mitochondrial subunit ribosomal RNA gene (mtSSU), and the largest subunit of RNA polymerase II (RPB1) sequences. The number in each node represents bootstrap support (BS) and posterior probability (PP). BS values of ≥70 and PP values of ≥0.95 are plotted on the branches. Scale in 0.02 substitutions per site.

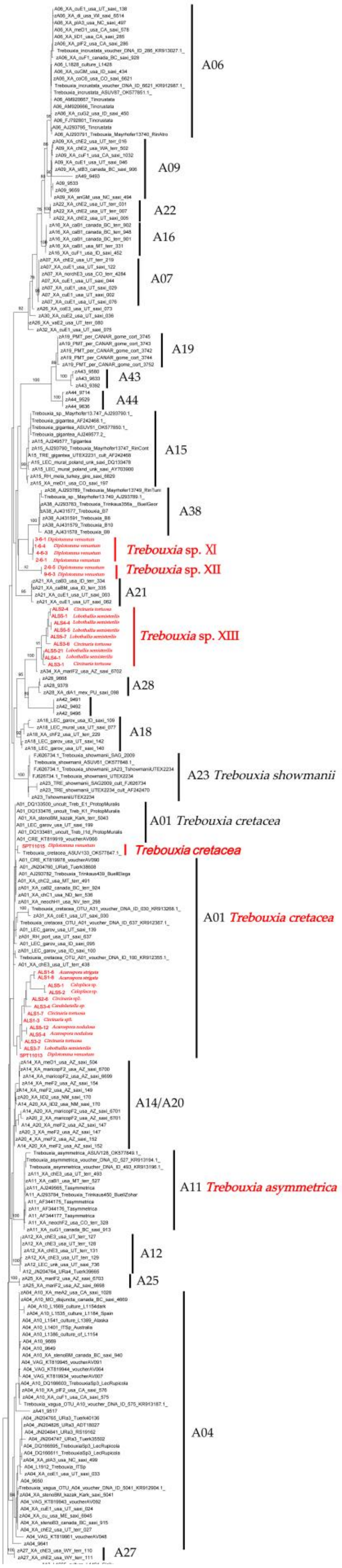
A06
A09
A22
A16
A07
A19
A43
A44
A15
A38
Trebouxia sp. XI
Trebouxia sp. XII
A21
Trebouxia sp. XIII
A28
A18
A23 Trebouxia showmanii
A01 Trebouxia cretacea
Trebouxia cretacea
A01 Trebouxia cretacea
A14/A20
A11 Trebouxia asymmetrica
A12
A25
A04
A27

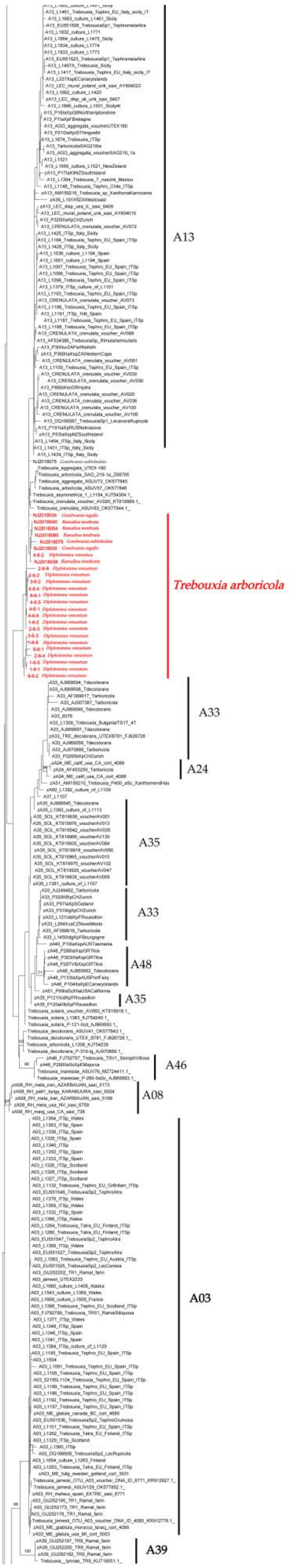
A13
Trebouxia arboricola
A33
A24
A35
A33
A48
A35
A46
A08
A03
A39

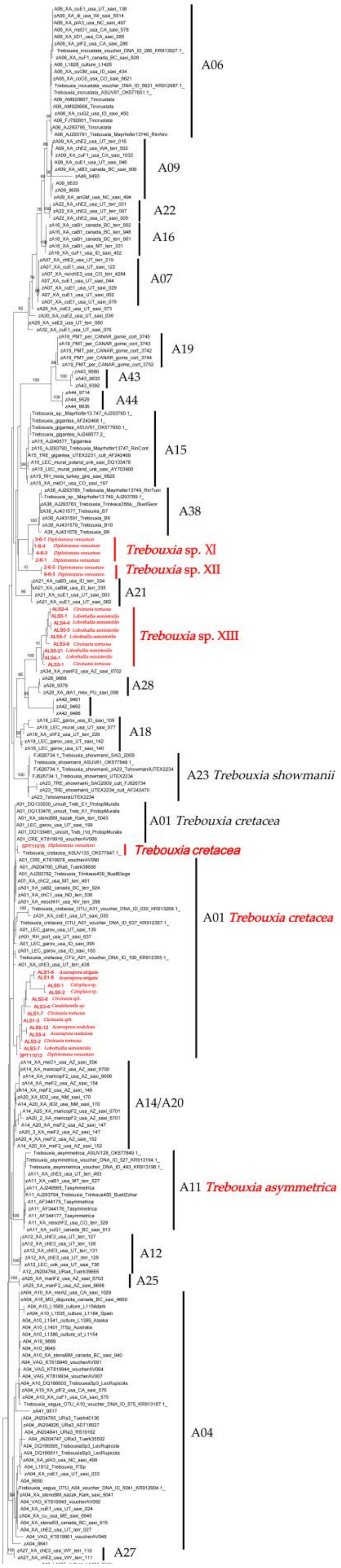
A06
A09
A22
A16
A07
A19
A43
A44
A15
A38
Trebouxia sp. XI
Trebouxia sp. XII
A21
Trebouxia sp. XIII
A28
A18
A23 Trebouxia showmanii
A01 Trebouxia cretacea
Trebouxia cretacea
A01 Trebouxia cretacea
A14/A20
A11 Trebouxia asymmetrica
A12
A25
A04
A27

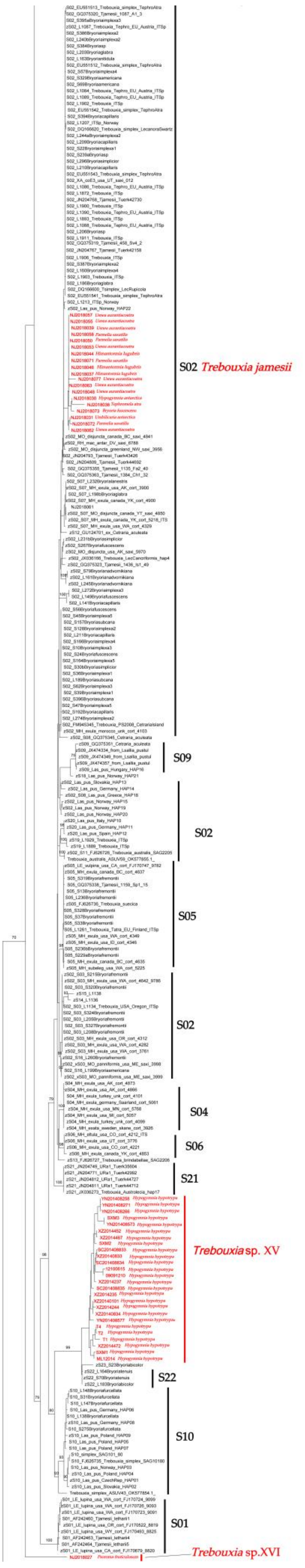
S02 Trebouxia jamesii
S09
S02
S05
S02
S04
S06
S21
Trebouxia sp. XV
S22
S10
S01
Trebouxia sp.XVI

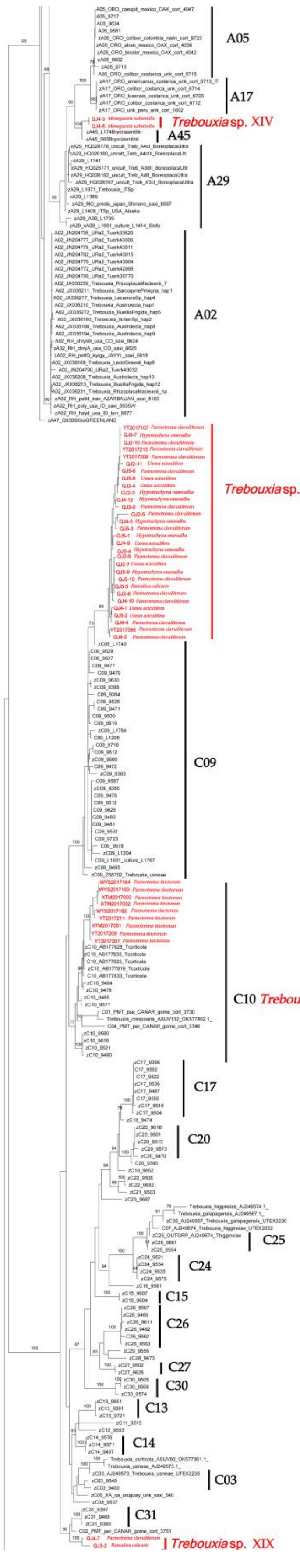

A05
A17
Trebouxia sp. XIV
A45
A29
A02
Trebouxia sp. XX
C09
C10 Trebouxia corticola
C17
C20
C25
C24
C15
C26
C27
C30
C13
C14
C03
C31
Trebouxia sp. XIX

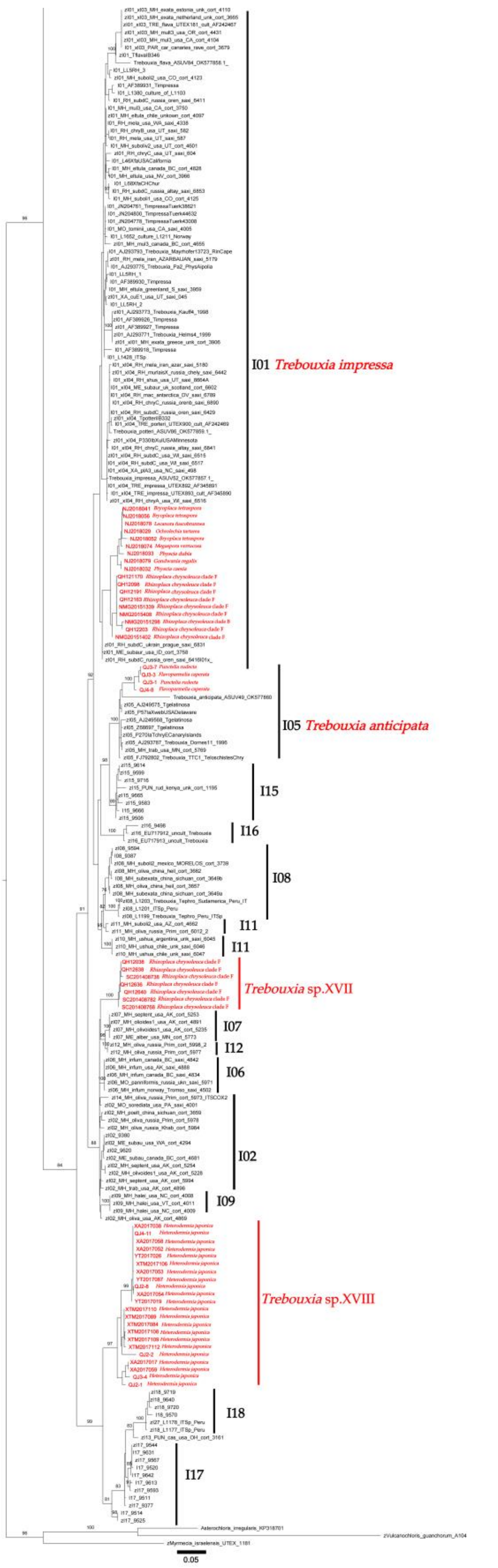

Figure S3. Phylogeny of lichen-forming algae *Trebouxia* species based on the Randomized Axelerated Maximum Likelihood (RAxML) analysis of a concatenated two-locus dataset including internal transcribed spacer region (ITS) and chloroplast ribulose-bisphosphate carboxylase-RuBisCO (*rbc*L) gene sequences, with reference sequences from the studies of [5,93–96] marked in black. The number in each

node represents bootstrap support (BS). BS values of ≥70 are plotted on the branches. Scale in 0.05 substitutions per site.

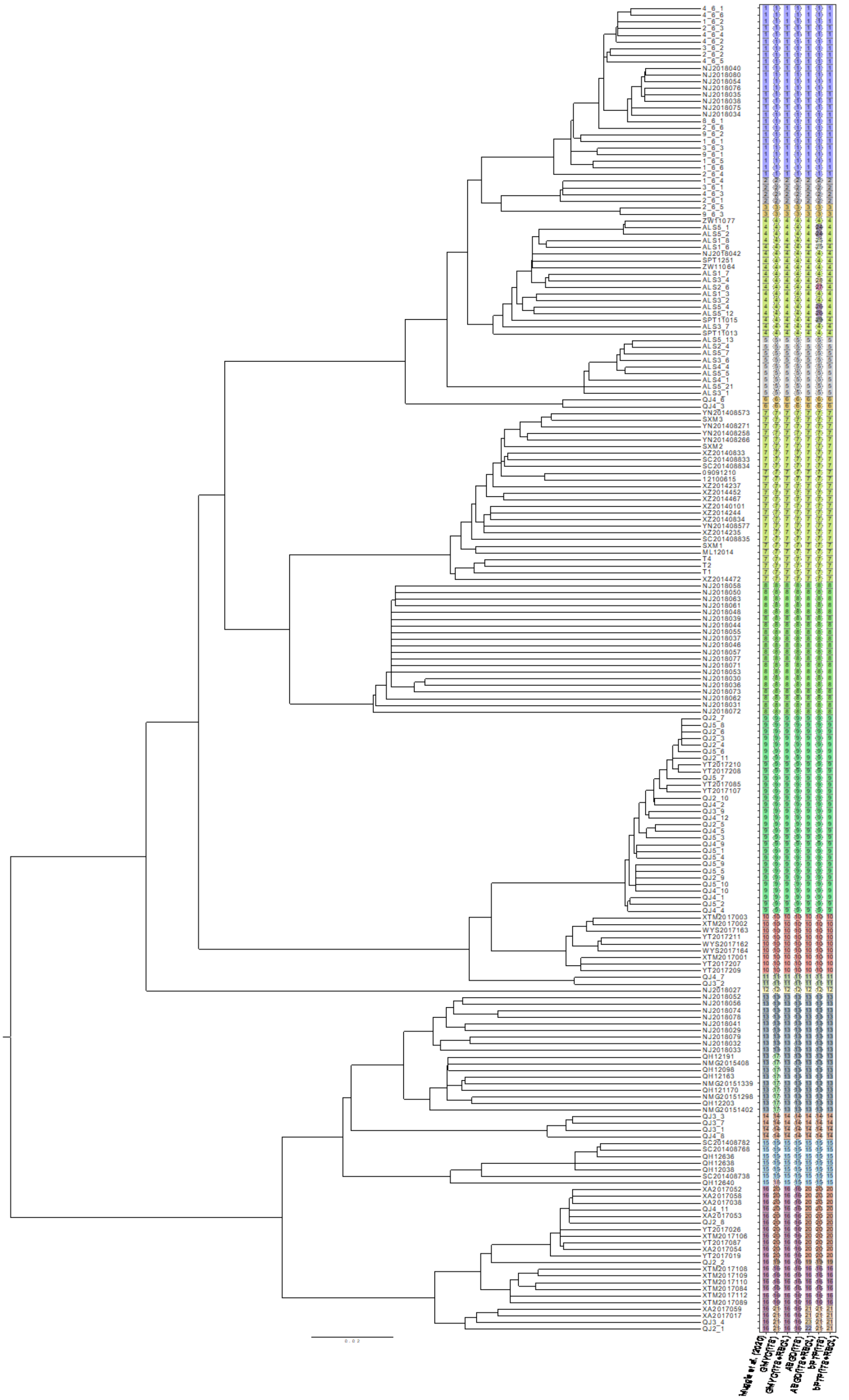

Figure S4. Comparison of different *Trebouxia* species delimitations resulting from three species delimitation approaches, i.e., Automatic Barcoding Gap Discovery (ABGD), a Bayesian implementation of the PTP (bPTP) [79], and the coalescent-based General Mixed Yule Coalescent (GMYC), along with the species scenarios proposed by [5]. The phylogeny was constructed by Randomized Axelerated Maximum

Likelihood (RAxML) analysis based on the combined internal transcribed spacer region (ITS) and chloroplast ribulose-bisphosphate carboxylase-RuBisCO gene (*rbc*L) gene sequences. Scale in 0.02 substitutions per site. The numbers in the seven columns represent the species numbers defined by different methods.

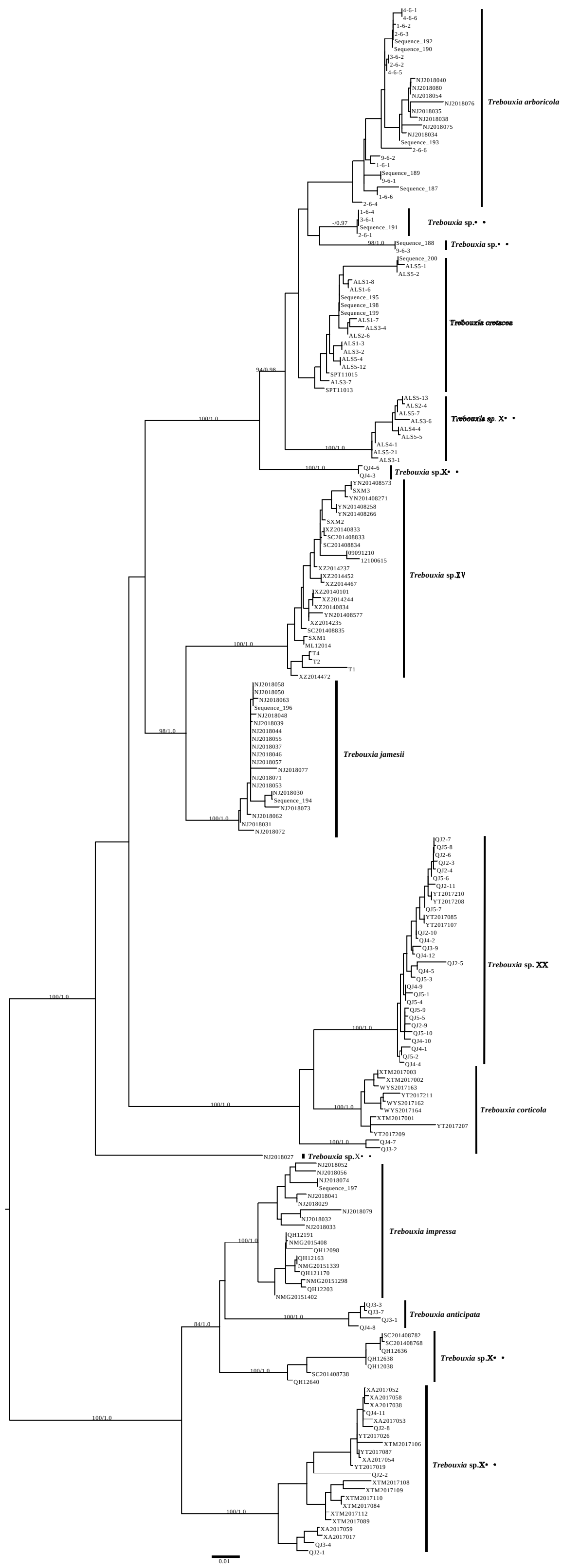

Figure S5. Phylogeny of lichen-forming algae *Trebouxia* species based on the Randomized Axelerated Maximum Likelihood (RAxML) analysis of a concatenated three-locus dataset including internal

transcribed spacer region (ITS), large nuclear subunit ribosomal RNA gene (nuSSU), and chloroplast ribulose-bisphosphate carboxylase-RuBisCO (*rbc*L) sequences. The number in each node represents bootstrap support (BS) and posterior probability (PP). BS values of ≥70 and PP values of ≥0.95 are plotted on the branches. Scale in 0.01 substitutions per site.

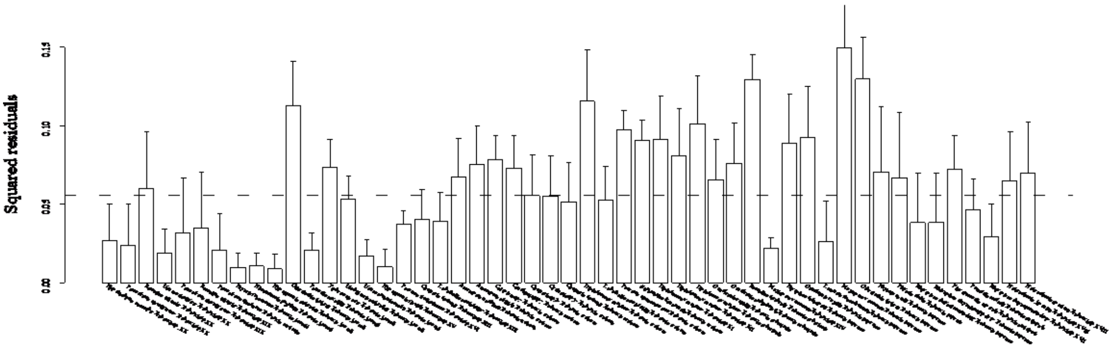

Figure S6. Boxplot of the jackknifed squared residuals, with upper 95% confidence intervals associated with each host–symbiont link from Procrustean Approach to Cophylogeny (PACo) analysis. Asterisks on the top of the bars indicate significant congruence, as supported by ParaFit.

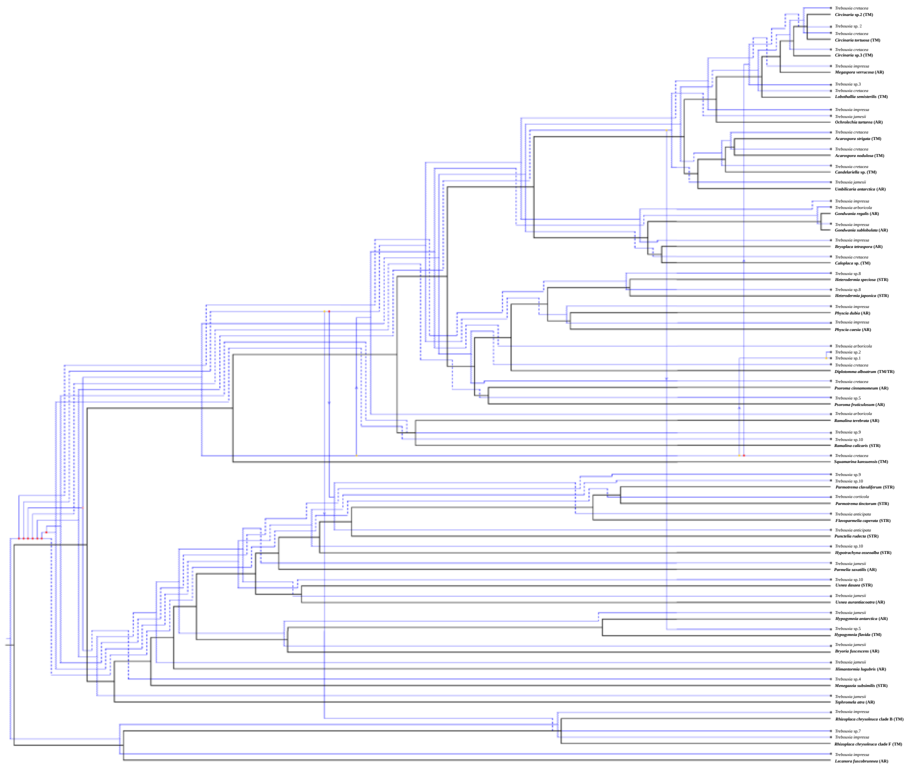

Figure S7. The least costly cophylogenetic scenario between lichen-forming algae (LFA) and their lichen-forming fungi (LFF) hosts, reconstructed using Jane 4.0. The cost regime settings were as follows: cospeciation = 0, duplication = 1, duplication with host switch = 2, losses = 1, and failures to diverge = −1, corresponding to cost regime E (Table 2). Black branches represent the LFF host phylogeny, and blue branches represent the LFA parasite phylogeny. The LFF names are in bold. Yellow and red solid circles represent duplications, dashed lines with purple circles represent losses, and dented lines with black asterisks represent failures to diverge. Habitat information is provided with the LFF species. AR: arctic/alpine or boreal zone, TM: temperate zone, STR: subtropical zone, TR: tropical zone.
