# Supplementary material for: Fungal–Algal Association Drives Lichens’ Mutualistic Symbiosis: A Case Study with Trebouxia-Related Lichens
Source: Plants (Basel). 2023 Sep 4;12(17):3172. doi: 10.3390/plants12173172 (PMC10490544; doi:10.3390/plants12173172)
Supplement: Supplementary file 1 [file plants-12-03172-s001.zip › Table S3.pdf]

Marker	Primer	Direction	Sequence (5'→3')	Annealing temperature (°C)	Reference
Mycobiont					
ITS	ITS4	Forward	TCCTCCGCTTATTGATATGC	55-60	[68]
	ITS5	Reverse	GGAAGTAAAAGTCGTAACAAGG		
LSU	LROR	Forward	ACCCGCTGAACTTAAGC	55-60	[67]
	LR5	Forward	ATCCTGAGGGAAACTTC		
	nu-LSU-155-5	Reverse	GGGTCCGAGTTGTAATTTGT		[57]
MtSSU	mrSSUl	Forward	AGCAGTGAGGAATATTGGTC'	52	[69]
	mrSSU3R	Reverse	ATGTGGCACGTCTATAGCCC		
RPB1	gRPB1-A	Forward	GA(G/T)TGTCC(T/G)GG(A/T)CATTTTG G	56	[66]
	fRPB-C	Revers	C(A/C/G/T)GC(A/T/G)AT(A/C/G/T)TC(A /G)TT(A/G)TCCAT(A/G)TA		[60]
	RPB1-HETER-F	Forward	CGGACCCATATCAAGCTGTT	55-50 (Touch down)	This study
	RPB1-HETER-R	Reverse	TGTCGCTACGTGAAATTGGA		
	RPB1-Rh-F	Forward	GCTTCATGACCAAAATCAAG		
	RPB1-Rh-R	Reverse	GAAACTGCAGTAGTTGTTCG		
Photobiont :					
ITS	nr-SSU-1780-59	Forward	CTGCGGAAGGATCATTGATTC	50	[63]
	nr-LSU-0012-39	Reverse	AGTTCAGCGGGTGGTCTTG		
	AL1500bf	Forward	GATGCATTCAACGAGCCTA	54	[59]
	LR3	Reverse	ACCTGCGGAAGGATCATT		[58]
rbcL	a-ch-rbcL-203 (f)	Forward	GAATCWTCWACWGGWACTTGGAC WAC	50	[61]
	a-ch-rbcL-991(r)	Reverse	CCTTCTARTTTACCWACAAC		
	PRASFl1	Forward	ATGGTTCCACAAACAGAAAC	47	[62]
	PRASR2	Reverse	CAGTGAAACCACCAGTTAAATAG		[65]
SSU	CV1	Forward	TACCTGGTTGATCCTGCCAGTAG	55	[64]
	CV2	Reverse	CCAATCCCTAGTCGGCATCGT		
	a(treb)-nu-SSU-0078-5′-mpn	Forward	CATGTCTAAGTATAAACTGCT	50	[56]
	a(treb)-nu-SSU-0803-3′-mpn	Reverse	TAGGCCAGAGTCCTATCGTGTTAT		

References

56. Dal Grande, F.; Beck, A.; Cornejo, C.; Singh, G.; Cheenacharoen, S.; Nelsen, M.P.; Scheidegger, C. Molecular phylogeny and symbiotic selectivity of the green algal genus *Dictyochloropsis* s.l. (Trebouxiophyceae): a polyphyletic and widespread group forming photobiont-mediated guilds in the lichen family Lobariaceae. *New Phytol.* **2014**, *202*, 455–470, doi:10.1111/nph.12678.
57. Döring, H.; Clerc, P.; Grube, M.; Wedin, M. Mycobiont-specific PCR primers for the amplification of nuclear ITS and LSU rDNA from lichenized ascomycetes. *Lichenologist* **2000**, *32*, 200–204, doi:10.1017/S0024282900000207.
58. FRIEDL, T.; ROKITTA, C. Species relationships in the lichen alga Trebouxia (Chlorophyta, Trebouxiophyceae): molecular phylogenetic analyses of nuclear-encoded large subunit rRNA gene sequences. *Symbiosis* **1997**, *23*, 125-148.

59. Helms, G.; Friedl, T.; Rambold, G.; Mayrhofer, H. Identification of photobionts from the lichen family Physciaceae using algal-specific ITS rDNA sequencing. *Lichenologist* **2001**, *33*, 73–86, doi:10.3732/ajb.89.4.688.
60. Matheny, P.B.; Liu, Y.J.; Ammirati, J.F.; Hall, B.D. Using RPB1 sequences to improve phylogenetic inference among mushrooms (Inocybe, Agaricales). *Am. J. Bot.* **2002**, *89*, 688–698.
61. Nelsen, M.P.; Plata, E.R.; Andrew, C.J.; Lücking, R.; Lumbsch, H.T. PHYLOGENETIC DIVERSITY OF TRENTEPOHLIALEAN ALGAE ASSOCIATED WITH LICHEN-FORMING FUNGI 1. *J. Phycol.* **2011**, *47*, 282–290, doi:10.1111/j.1529-8817.2011.00962.x.
62. Nozaki, H.; Itoh, M.; Sano, R.; Uchida, H.; Watanabe, M.M.; Kuroiwa, T. Phylogenetic relationships within the colonial Volvocales (Chlorophyta) inferred from rbcL gene sequence data. *J. Phycol.* **1995**, *31*, 970–979, doi:10.1111/j.0022-3646.1995.00970.x.
63. Piercey-Normore, M.D.; DePriest, P.T. Algal switching among lichen symbioses. *Am. J. Bot.* **2001**, *88*, 1490–1498, doi:10.2307/3558457.
64. Sawayama, S.; Inoue, S.; Yokoyama, S.y. Phylogenetic position of *Botryococcus braunii* (Chlorophyceae) based on small subunit ribosomal RNA sequence data 1. *J. Phycol.* **1995**, *31*, 419–420, doi:10.1111/j.0022-3646.1995.00419.x.
65. Sherwood, A.R.; Garbary, D.J.; Sheath, R.G. Assessing the phylogenetic position of the Prasiolales (Chlorophyta) using rbc L and 18S rRNA gene sequence data. *Phycologia* **2000**, *39*, 139–146, doi:10.2216/i0031-8884-39-2-139.1.
66. Stiller, J.W.; Hall, B.D. The origin of red algae: implications for plastid evolution. *Proc. Natl. Acad. Sci. U.S.A.* **1997**, *94*, 4520–4525, doi:10.1073/pnas.94.9.4520.
67. Vilgalys, R.; Hester, M. Rapid genetic identification and mapping of enzymatically amplified ribosomal DNA from several *Cryptococcus* species. *J. Bacteriol.* **1990**, *172*, 4238–4246, doi:10.1128/jb.172.8.4238-4246.1990.
68. White, T.J.; Bruns, T.; Lee, S.; Taylor, J. Amplification and direct sequencing of fungal ribosomal RNA genes for phylogenetics. In *PCR Protocols: - A Guide to Methods and Applications*; White, T.J., Bruns, T., Lee, S., Eds.; Academic Press, 1990; pp. 315–322. doi:10.1016/B978-0-12-372180-8.50042-1.
69. Zoller, S.; Scheidegger, C.; Sperisen, C. PCR primers for the amplification of mitochondrial small subunit ribosomal DNA of lichen-forming Ascomycetes. *Lichenologist* **1999**, *31*, 511–516, doi:10.1006/lich.1999.0220.
